# CFD and experimental data of closed-loop wind tunnel flow

**DOI:** 10.1016/j.dib.2016.02.033

**Published:** 2016-02-20

**Authors:** John Kaiser Calautit, Ben Richard Hughes

**Affiliations:** Department of Mechanical Engineering, University of Sheffield, Sheffield S10 2TN, UK

**Keywords:** Aerodynamics, Computational modelling, Flow quality, Wind engineering, Wind tunnel

## Abstract

The data presented in this article were the basis for the study reported in the research articles entitled ‘A validated design methodology for a closed loop subsonic wind tunnel’ (Calautit et al., 2014) [1], which presented a systematic investigation into the design, simulation and analysis of flow parameters in a wind tunnel using Computational Fluid Dynamics (CFD). The authors evaluated the accuracy of replicating the flow characteristics for which the wind tunnel was designed using numerical simulation. Here, we detail the numerical and experimental set-up for the analysis of the closed-loop subsonic wind tunnel with an empty test section.

**Specifications Table**

**Table:** 

Subject area	*Environmental Science*
More specific subject area	*Aerodynamics, Airflow, Computational modelling, Wind engineering*
Type of data	*Tables, graphs, figure, Computer aided design (CAD) geometry*
How data was acquired	*FLUENT 14.0 for numerical analysis, Hot-wire anemometer (Testo 425) for airflow measurement*
Data format	*Raw data and analysed*
Experimental factors	*Controlled wind tunnel velocity inside a closed-loop, subsonic wind tunnel. Numerical wind tunnel geometry and boundary conditions were fully based on the actual closed-loop wind tunnel.*
Experimental features	*The experimental tests were conducted with an empty test section. The airflow velocities were measured at the inlet and outlet of the wind tunnel test section. Airflow velocity measurements were performed along two vertical lines located in the inlet and outlet of the test-section), at intervals of 0.025 m. The measurements were recorded when the temperature of the wind tunnel became stabilised at an ambient temperature of 298 K. For the numerical model, three-dimensional Reynolds-averaged Navier-Stokes (RANS) equations and the continuity equation were solved using FLUENT 14.0 which employed the control-volume technique and the Semi-Implicit Method for Pressure-Linked Equations (SIMPLEC) velocity-pressure coupling algorithm with the second order upwind discretization. Standard k-epsilon turbulence model.*
Data source location	*Sheffield, United Kingdom*
Data accessibility	*Data is with this article*

**Value of the data**

• The numerical and experimental data files can be used by wind tunnel designers, engineers and researchers when validating the prediction of their closed-loop wind tunnel models (theoretical, computational methods, etc.) with an empty test section.
• The numerical and experimental data can be used to test different turbulence models, boundary conditions, mesh design, discretisation scheme, steady-state and transient simulations etc.
• The value of the data is its use in improving the comparability between the results of other researcher׳s models by providing a common benchmark.
• The data can be used for training of CFD users and contribute to an overall improvement of the prediction accuracy of CFD modelling of wind tunnels.
• The data can be used to explore different optimisation of the full closed-loop wind tunnel design and also its separate components.

## 1 Data

**Fig. 1:**
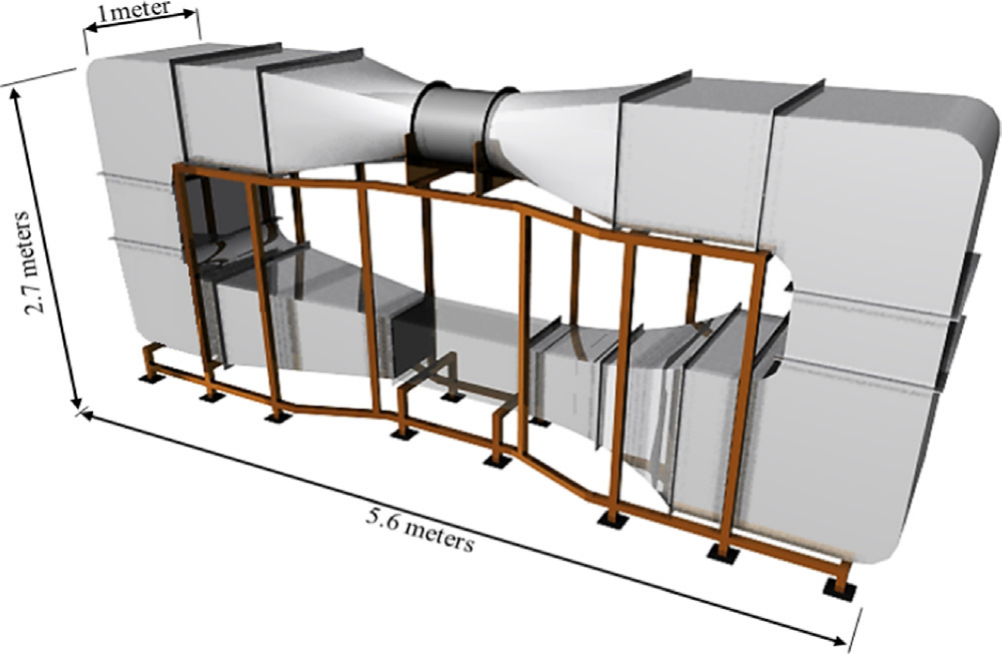
Schematic of the closed loop subsonic wind tunnel facility.

**Fig. 2:**
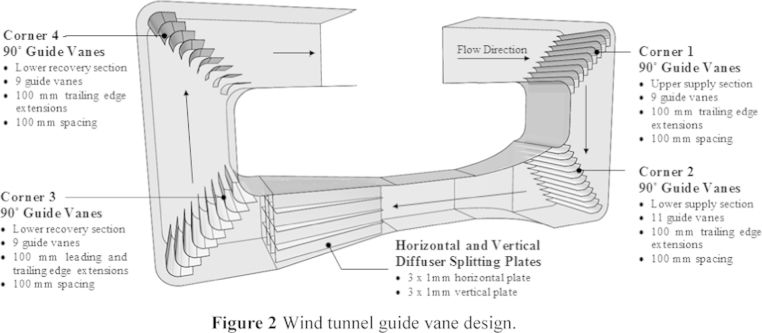
Wind tunnel guide vane design.

The data presented in this article is based on the investigation into the simulation of the airflow in a wind tunnel (Fig. 1) which was conducted using Computational Fluid dynamics (CFD) modelling and experimental testing [1]. The closed-loop wind tunnel is used for a wide range of applications [2], [3], [4], [5], [6], [7], [8], [9], [10], [11], [12], [13], [14]. The data used for the investigation of four types of guide vane (GV) configurations (no-GV, upstream-GV, downstream-GV and upstream-downstream-GV) are shared in the article and the geometry files which were created using SolidEdge software are also provided (Supplementary 2) to save time and effort (Fig. 2).

**Fig. 3:**
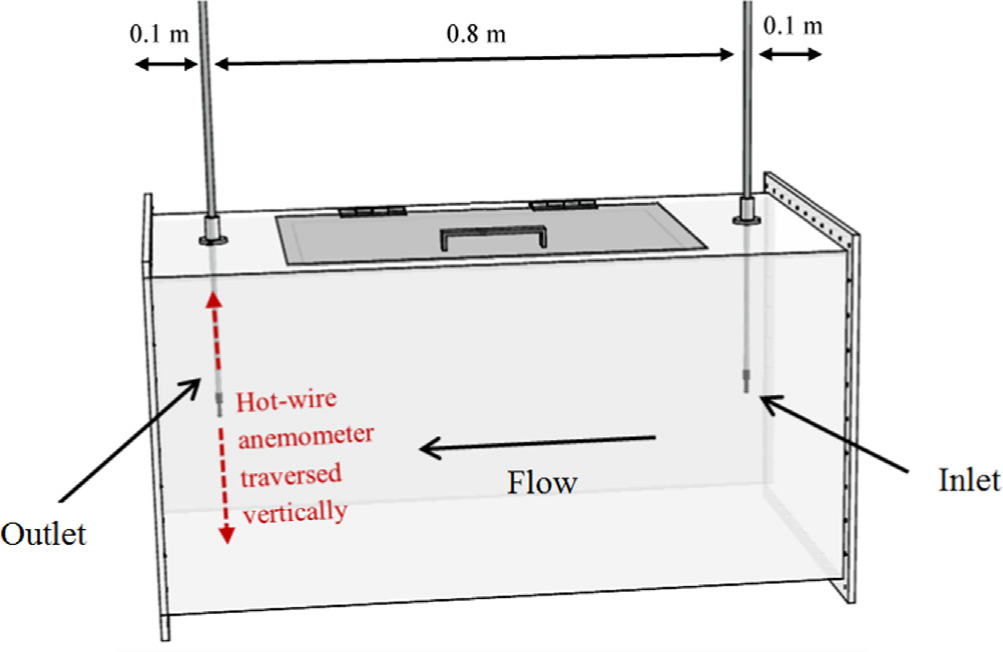
Location of measurement points inside the test section.

The data shared in this article are presented in the supplementary file in the following order:

**Data for the numerical analysis of wind tunnel with no guide vanes** (Supplementary Table 1) – The data presented in this file are the vertical airflow velocity profiles (Fig. 3) inside the wind tunnel without guide vanes.

**Data for the numerical analysis of wind tunnel with upstream guide vanes** (Supplementary Table 2) – The data presented in this file are the vertical airflow velocity profiles inside the wind tunnel with upstream guide vanes.

**Data for the numerical analysis of wind tunnel with downstream guide vanes** (Supplementary Table 3) – The data presented in this file are the vertical airflow velocity profiles inside the wind tunnel with downstream guide vanes.

**Data for the numerical analysis of wind tunnel with upstream and downstream guide vanes** (Supplementary Table 4) – The data presented in this file are the vertical airflow velocity profiles inside the wind tunnel with both upstream and downstream guide vanes.

**Data for the experimental analysis of wind tunnel with upstream and downstream guide vanes** (Supplementary Table 5) – This data file presents the measurements of the vertical airflow velocity profile at the inlet and outlet of the test section, at intervals of 0.025 m.

**Data for the comparison analysis of numerical and experimental results** (Supplementary Table 6) – This data file presents a the CFD and experimental results of the inflow and outflow of the test section of the wind tunnel with upstream and downstream guide vanes.

**Wind tunnel geometry** (Supplementary 2) **–** The data files are full scale CAD models of the closed-loop wind tunnel geometry. The CAD models are available in IGS format and can be directly imported to most commercial CFD software such as FLUENT and CFX. There are four types; no-GV, upstream-GV, downstream-GV and upstream-downstream-GV. The actual wind tunnel is based on the “upstream and downstream guide vanes” model.

## 2 Experimental design, material and methods

### 2.1 Numerical design and data collection method.

The commercial ANSYS Fluent14 numerical code was used for predicting the flow characteristics inside the wind tunnel. A full-scale CFD model of the entire wind tunnel was considered in the study [1]. The numerical code was used to solve the Reynolds averaged Navier–Stokes equation (RANS simulation) which employed a control-volume-based technique. The standard k-ε turbulence model was used for defining the turbulence kinetic energy and dissipation rate within the model second-order upwind scheme was used to discretised all the transport equations. The numerical code used the semi-implicit method for pressure-linked equations (SIMPLE) algorithm for the velocity–pressure coupling of the computation. The rationale behind choosing the k-epsilon model was the findings of our previous work [1], which showed its capabilities in predicting the wind tunnel flows. The selected resolution of the grid was based on the grid-sensitivity analysis. Structured mesh was used for sections dominated by one-dimensional flow and tetrahedral mesh for sections with three-dimensional flows. The complete meshed model comprised of 4.2 million elements. The computations were performed using parallel processing on a workstation with one Intel Xeon 2.1 GHz processor and 16 GB Fully Buffered DDR2. The simulations were completed after additional iterations showed no further variation in the velocity results. The airflow velocity data were collected by plotting three vertical lines (each with 42 data points equally spaced across the line) inside the test section; inflow (0.1 m from inlet), middle (center of test section) and outflow (0.1 m from outlet).

### 2.2 Experimental design and data collection method

The wind tunnel had an overall length of 5.6 m with a 0.5 m(*W*)×0.5 m(*H*)×1 m(*L*) test section. The tunnel operated as closed-loop; air that passed through the test section was drawn back into the fan and recirculated into the test section repeatedly. All of the wind tunnel components were manufactured from steel except for the circular to rectangular duct (neoprene), test section (perspex glass) and some parts of the fan. The walls of the test section was made of clear perspex material to allow the accurate positioning of the hot-wire anemometer sensors inside the space. The fan which drives the wind tunnel systems was a 0.7 m diameter 2.1 kW axial variable-revolution fan. Full details of the design, specification and operation of the wind tunnel are available in [1].

Fig. 3 shows the experimental design for measuring the vertical airflow velocity profile at the inlet and outlet of the test section using a Testo 425 hot-wire anemometer. The measurements were carried out in the same locations as the numerical analysis for direct comparison. The hot-wire probe was accurately positioned within the test section when collecting velocity measurements by using the telescopic arm of the probe to determine the length of the probe and holes drilled into the test section which matched the measurement points. The measurements were recorded when the temperature of the wind tunnel became stabilised at an ambient temperature of 298 K. The average was calculated for the period of two minutes, while the results and start or finish times were recorded. In addition, multiple measurements (4 times) were taken for each point to increase the certainty of the measurement. The uncertainties associated with the air velocity readings, the hot-wire probe gave velocity measurements with uncertainty of ±1.0% rdg. at speeds lower than 8 m/s and uncertainty of±0.5% rdg. at higher speeds (8–20 m/s).

## References

[1] Calautit J.K., Chaudhry H.N., Hughes B.R., Sim L.F. (2014). A validated design methodology for a closed-loop subsonic wind tunnel. J. Wind Eng. Ind. Aerodyn..

[2] Calautit J.K., O’Connor D., Hughes B.R. (2014). Determining the optimum spacing and arrangement for commercial wind towers for ventilation performance. Build. Environ..

[3] Sofotasiou P., Calautit J.K., Hughes B.R., O’Connor D. (2016). Towards an integrated computational method to determine internal spaces for optimum environmental conditions. Comput. Fluids.

[4] Calautit J.K., Hughes B.R., Shahzad S.S. (2015). CFD and wind tunnel study of the performance of a uni-directional wind catcher with heat transfer devices. Renew. Energy.

[5] Calautit J.K., Hughes B.R. (2016). A passive cooling wind catcher with heat pipe technology: CFD, wind tunnel and field-test analysis. Appl. Energy.

[6] Calautit J.K., Hughes B.R. (2014). Wind tunnel and CFD study of the natural ventilation performance ofa commercial multi-directional wind tower. Build. Environ..

[7] Calautit J.K., Hughes B.R. (2014). Measurement and prediction of the indoor airflow in a room ventilated with a commercial wind tower. Energy Build..

[8] Calautit J.K., Chaudhry H.N., Hughes B.R. (2015). Wind tunnel data of the analysis of heat pipe and wind catcher technology for the built environment. Data Brief.

[9] Chaudhry H.N., Hughes B.R., Calautit J.K. (2015). Sim LF,CFD and experimental study on the effect of progressive heating on fluid flow inside a thermal wind tunnel. Computation.

[10] Calautit J.K., O׳Connor D., Hughes B.R. (2016). A natural ventilation wind tower with heat pipe heat recovery for cold climates. Renew. Energy.

[11] Calautit J.K., Hughes B.R. (2014). Integration and application of passive cooling within a wind tower for hot climates. HVACR Res..

[12] Calautit J.K., O’Connor D., Sofotasiou P., Hughes B.R. (2015). CFD simulation and optimisation of a low energy ventilation and cooling system. Computation.

[13] O’Connor D., Calautit J.K., Hughes B.R. (2014). A study of passive ventilation integrated with heat recovery. Energy Build..

[14] O’Connor D., Calautit J.K., Hughes B.R. (2015). Effect of rotation speed of a rotary thermal wheel on ventilation supply rates of wind tower system. Energy Procedia.

